# The Impact of Hyperbaric Oxygen Therapy in Liver Donors Before Controlled Circulatory Death on Graft Function Recovery: A Pilot Study

**DOI:** 10.3389/ti.2025.15019

**Published:** 2025-11-27

**Authors:** Morgan Caplan, Juliette Philipon, Sébastien Dharancy, Emmanuel Boleslawski, Guillaume Strecker, Bruno Mourvillier, Erika Parmentier-Decrucq, Vincent Dupont

**Affiliations:** 1 Médecine Intensive et Réanimation Polyvalente, Centre Hospitalier Universitaire de Reims, Reims, France; 2 Pôle de Réanimation, Hôpital Salengro, Lille, France; 3 Hépato-Gastro-Entérologie, Centre Hospitalier Universitaire de Lille, Lille, France; 4 Département de Chirurgie Digestive et Transplantation, Université de Lille, Lille, France; 5 Pôle Anesthésie-Réanimation, Hôpital Salengro, Lille, France; 6 Néphrologie, Centre Hospitalier Universitaire de Reims, Reims, France

**Keywords:** liver transplantation, hyperbaric oxygen therapy, ischemia reperfusion injury, donation after circulatory death, early allograft dysfunction

Dear Editors,

Hyperbaric oxygen therapy (HBO) is a specialized treatment modality that involves the administration of 100% oxygen at pressures exceeding atmospheric levels (ATA) [1]. Currently, there is no data on the impact of HBO prior to organ donation on graft function recovery. However, the combination of elevated oxygen levels and increased pressure may influence liver function, especially in the context of ischemia-reperfusion (IR) injury.

The objective of this study was to evaluate graft function recovery in liver transplant recipients from donors who received HBO before organ procurement compared to recipients from untreated donors.

We conducted a retrospective study at a high transplant-volume university center in Lille, France, where HBO therapy is commonly used in life-threatening hanging cases, occasionally leading to controlled donation after circulatory death (DCD) procedures. We included all liver transplantations (LT) from DCD donors over the period 1 January 2018, to 31 December 2023, and excluded donors/recipients aged <18 years or with missing data.

Recipient outcomes were analyzed based on the HBO treatment performed before the liver donation procedure. Each HBO session involved a 15-min pressure rise from 1 to 2.5 ATA, maintaining this pressure for 90 min with an inspired oxygen fraction of 100%, followed by a 15-min decompression period [1].

The French national protocol for DCD includes systematic normothermic regional perfusion (NRP). Transaminase kinetics and maximum values recorded during NRP are considered markers of indirect viability of the liver graft. Graft biopsy was systematically performed. All LT procedures used standardized anesthetic and surgical protocols with the side-to-side cavocaval technique. The institutional immunosuppression protocol consists of an initial triple-immunosuppressive regimen (calcineurin inhibitors, corticosteroids, and anti-metabolites).

Donor and recipient characteristics were collected from medical records by a member of the intensive care unit (ICU) and a hepatologist, respectively, then anonymized before analysis.

The primary outcome was the occurrence of early allograft dysfunction (EAD) according to the Olthoff definition based on the occurrence of at least one of the following criteria: bilirubin concentration ≥10 mg/dL on post-operative day (POD) 7, INR value ≥1.6 POD, and AST or ALT >2.000 U/L within the first seven POD [2]. We also studied the model of early allograft function (MEAF), which allows a quantitative assessment of allograft function within the first three POD.

This study was conducted in accordance with current French legislation (CNIL and MR004).

Data were graphed and statistics were calculated using GraphPad Prism, version 9.5.1 (GraphPad Software). Quantitative and qualitative data were compared using the Mann–Whitney test and Fisher exact test or χ^2^ test with Yates correction, respectively. The Benjamini-Hochberg procedure was applied to account for multiplicity. Statistical significance was set at *P* < 0.05.

During the study period, 554 LTs were performed including 68 donors with DCD. Among the latter, 5 and 4 LT were excluded due to missing recipient and donor data (organs retrieved or transplanted in a different center). Thus, we included 59 LT in our study, of which 25 (42.3%) involved donors exposed to HBO before donation (Table 1). All HBO-treated donors underwent a total of five sessions within the first 48 h of ICU stay, with a median time between the last HBO session and donation procedure of 6 (5–8) days.

**TABLE 1:** Donors and recipients’ characteristics.

Variables^a^	HBO - (n = 34)	HBO + (n = 25)	Univariate analysis
			*P value*
Donors’ baseline characteristics			
Age, *years*	56 (47–66)	44 (31–50)	**<0.001**
Male, *n (%)*	26 (76.5)	22 (88)	0.33
BMI, *kg/m* ^*2*^	28 (25–30)	24 (22–27)	**0.001**
Smoke, *n (%)*	14 (41.2)	11 (44)	1
Alcohol, *n (%)*	7 (20.6)	9 (36)	0.31
Hypertension, *n (%)*	12 (35.3)	4 (16)	0.14
Diabetes, *n (%)*	4 (11.8)	0 (0)	0.13
Cause of admission			
Suicide by hanging *n (%)*	1 (2.9)	25 (100)	**<0.001**
Stroke, *n (%)*	8 (23.5)	0 (0)	**0.02**
SAPSII score	55 (47–65)	55 (47–61)	0.90
Cardiac arrest, *n (%)*	20 (58.8)	24 (96)	**0.002**
No flow, *min*	5 (0–7)	20 (15–30)	**<0.001**
Low flow, *min*	23 (19–30)	20 (12–24)	**0.03**
Electric shock, *n (%)*	12 (35.3)	2 (8)	**0.03**
Adrenaline, *mg*	2 (1–4)	1 (0–2)	**0.01**
Admission lactate level, *mmol/L*	6.3 (3.6–7.3)	4 (2.9–6.8)	0.10
Biological parameters before circulatory arrest			
PT, %	79 (70–83)	84 (71–96)	0.19
Total bilirubin, umol/L	5 (4–7)	5 (3–7)	0.40
AST, UI/L	52 (30–79)	58 (42–82)	0.35
ALT, UI/L	41 (23–74)	62 (37–101)	**0.04**
Maastricht 3 procedure characteristics			
Circulatory arrest time, *min*	18 (15–22)	17 (15–19)	0.52
Total WIT, *min*	32 (27–42)	28 (25–34)	0.11
Functional WIT, *min*	22 (20–29)	20 (18–25)	0.08
Biological parameters during NRP			
AST peak, UI/L	55 (30–101)	65 (50–85)	0.18
ALT peak, UI/L	35 (23–86)	53 (41–95)	**0.02**
Macrovacuolar steatosis >5%, *n (%)*	20 (58.8)	11 (44)	0.61
Recipients’ baseline characteristics			
Age, *years*	59 (54–61)	57 (49–61)	0.56
Male, *n (%)*	24 (66.7)	20 (74.1)	0.55
BMI, *kg/m* ^*2*^	28 (25–30)	28 (25–32)	0.69
Causal liver disease			
Alcoholic, *n (%)*	21 (58.3)	21 (77.8)	0.08
Viral, *n (%)*	5 (13.9)	5 (18.5)	0.73
Child pugh score	6 (5–8)	7 (5–9	0.40
MELD score	10 (7–14)	11 (8–16)	0.33
Cold ischemia time, *min*	278 (241–329)	265 (225–291)	0.22
Liver recipients and early graft outcomes			
AST or ALT >2000UI/L within the first 7 POD, *n (%)*	6 (17.6)	3 (12)	0.52
Bilirubin ≥10 mg/dL on POD 7, *n (%)*	15 (44.1)	7 (28)	0.19
INR ≥1.6 on POD 7, *n (%)*	0 (0)	0 (0)	1
Early graft dysfunction (olthoff), *n (%)*	20 (58.8)	9 (36)	0.08
MEAF score	5.1 (4.2–6.0)	5.0 (3.6–5.4)	0.36
ICU stay, *days*	9 (7–12)	10 (8–12)	0.34
Hospital stay, *days*	13 (10–17)	13 (10–16)	0.97
New liver transplantation within 1 year, *n (%)*	2 (5.9)	1 (4)	1
Death within 1 year, *n (%)*	2 (5.9)	2 (8)	1

^a^ Data are reported as numbers (percentages) or medians (interquartile ranges). HBO, hyperbaric oxygen therapy; BMI, body mass index; SAPSII, simplified acute physiologic score II; PT, prothrombin time; WIT, warm ischemia time; NRP, normothermic regional perfusion; AST, aspartate aminotransferase; ALT, alanine aminotransferase; POD, post operative day; MEAF, model for early allograft function; MELD, model for end-stage liver disease; INR, international normalized ratio.

Bold values indicate statistically significant p-values (p < 0.05).

The causes of death differed between the two groups and reflected local practices (as HBO is performed in cases of suicide by hanging). Donors from the HBO group had a lower BMI than the controls. Admission severity was similar between the two groups. Compared with controls, recipients from the HBO donor group showed higher prothrombin time from POD 2 to 14 as well as lower bilirubin values on POD 14 and 1 month after LT.

To our knowledge, this is the first study to assess HBO as a preconditioning intervention for liver graft outcomes. Our primary finding was that HBO treatment did not adversely affect the graft or recipient outcomes. These results suggest the feasibility and safety of HBO administration in ICU patients who later become organ donors. Interestingly, despite the limited sample size, we observed a trend toward reduced EAD occurrence in recipients from HBO-treated donors compared to controls, alongside a significant improvement in prothrombin time and bilirubin levels post-LT. Furthermore, this trend toward improved hepatic recovery was observed when HBO was not intentionally used as a preconditioning therapy [1], which led to a significant delay between HBO treatment and the procurement procedure.

From a mechanistic perspective, HBO may exert its effects through the reduction of ischemia-reperfusion injury, attenuation of oxidative stress, and modulation of inflammatory pathways, as previously suggested in experimental models [3–5]. In addition, HBO has been shown to promote hepatocyte regeneration and improve microcirculatory function, which could explain the favorable trends we observed in graft function recovery. Further mechanistic studies are warranted to delineate these potential pathways in the clinical transplantation setting.

This study had several limitations. First, its small sample size and retrospective design hindered the feasibility of multivariate analysis to account for confounding variables. Hence, the substantial baseline differences between the two groups of donors restrict the strength of our conclusions. Accordingly, our results should be interpreted as exploratory and hypothesis-generating, providing a rationale for larger prospective multicenter studies. Second, there were notable differences in donor characteristics between the two groups, though the similar admission severity score strengthens the observed trends. Finally, the study design did not allow us to differentiate whether the potential benefits of HBO on early graft function were due to post-exposure effects following cardiac arrest or a preconditioning effect before organ procurement.

Taken together, our results primarily support the feasibility and safety of hyperbaric oxygen therapy in potential liver donors. Rather than demonstrating a proven benefit, these preliminary findings should be regarded as hypothesis-generating and provide a rationale for future prospective and multicenter investigations.

Overall, our findings highlight favorable trends in post-transplant hepatic function and support the feasibility and safety of HBO in this donor population, while underscoring the need for larger, prospective studies to confirm these preliminary observations.

## Data Availability

The raw data supporting the conclusions of this article will be made available by the authors, without undue reservation.

## Abbreviations

ATA, Atmospheric pressure; DCD, Donation after circulatory death; EAD, Early allograft dysfunction; HBO, Hyperbaric oxygen therapy; ICU, Intensive care unit; IR, Ischemia-reperfusion; LT, Liver transplantation; MEAF, Model of early allograft function; MELD, Model for end-stage liver disease score; NRP, Normothermic regional perfusion; POD, Post-operative days.
